# HSV-2 Seroepidemiology and Risk Factors among Iranian Women: A Time to New Thinking

**Published:** 2011-11-01

**Authors:** S Asgari, L Chamani-Tabriz, S Asadi, F Fatemi, H Zeraati, M M Akhondi, A Shahnazi

**Affiliations:** 1 International Campus, Tehran University of Medical Sciences, Kish, Iran; 2 Reproductive Biotechnology Research Center, Avicenna Research Institute, ACECR, Tehran, Iran; 3 Infectious Diseases and Tropical Medicine Research Center, Shahid Beheshti University of Medical Sciences, Tehran, Iran; 4 Department of Epidemiology and Biostatistics, School of Public Health, Tehran University of Medical Sciences, Tehran, Iran

**Keywords:** Genital, Herpes Simplex Virus, Female, Behavior, Iran

## Abstract

**Background:**

Genital herpes is a common sexually transmitted disease in many developed and developing countries mostly caused by Herpes simplex virus type 2 (HSV-2). This study determines the prevalence of HSV-2 infection between two groups of women with high and low risk behaviors.

**Methods:**

In this seroepidemiologic study, 362 women attending obstetrics and gynecology clinics as low risk group and 156 prisoners and drop in center resident women in Tehran as high risk group were enrolled. HSV infection was identified by serologic tests on blood samples.

**Results:**

The prevalence of IgG antibody in high risk group was significantly more than low risk women (26.3% vs. 2.5%). The prevalence of IgM antibody in high risk group was less than low risk group (3.8% vs. 7.1%) but the difference was not statistically significant. In high risk group, there was significant association between positive IgG and anal/oral sex, use of condom, smoking and drug addiction as well as genital pain, burning, itching, ulcer, dysuria, and history of genital infection. In low risk group, association between positive IgM and IgG test results and risky behaviors were not significant. There was significant association between IgM and genital itching, rash, and ulcer.

**Conclusion:**

Relatively high seroprevalence of anti-HSV-2 IgG and high frequency of genital Herpes among high risk women necessitates regular screening and safe sex education programs. Moreover, risk of acute infection in this group should not be ignored and its distribution in Iranian population should be alarmingly concerned.

## Introduction

Genital Herpes is a common sexually transmitted disease (STD) in many developed and developing countries. It is caused by the Herpes simplex virus type 1 (HSV-1) or type 2 (HSV-2) but the most genital Herpes is caused by HSV-2. After initial infection, the virus can reside as life-long virus and remains latent until opportunity for recurrence, thus genital Herpes is a recurrent, incurable viral disease.[1] Periodic recurrent infections are associated with viral shedding at the site of primary infection.[2] The primary and subsequent recurrent infections are affected by factors such as surgical operation, stress and immune suppression.[3] The majority of both primary and recurrent infections are asymptomatic diseases[4] however, in symptomatic cases lesions are very painful[5] and obviously affect the quality of life in patients.[6][7] Because of asymptomatic nature of disease, Herpes simplex viruses will be easily spread in population and is a suitable marker to evaluate the sexual behaviors.[5]

Genital Herpes is associated with psychological morbidity,[8] so the social stigma attached to this disease has been investigated and its impact on patients has been emphasized in many studies.[9][10] Nowadays, the synergistic interaction between HSV-2 and transmission of HIV is a risk factor associated with a twoto three-fold increased risk of HIV acquisition.[11][12][13][14]

Estimating the global burden of diseases is considerable to recognize the scale of an epidemic, encourage governments and organizations for investing in disease control and proper distribution of resources to those most affected.[15] The prevalence of HSV-2 in different countries was collected by systematic review study in 2002,[16] then in 2005,[17] and finally the universal genital Herpes outbreak by age and gender has been reported in 2008.[18] Although the prevalence in the Middle East and North Africa has been reported lower than other regions,[19] but the statistical data in Iran are not presented in these studies which requires a prevalence study in our country.

In attention to the prevalence of infection in developing countries, HSV-2 transmission through sexual contacts, asymptomatic cases of disease which caused rapid infection spread, incurable and recurrent nature of HSV-2 illness, necessitate genital Herpes simplex screening programs. Therefore, this study aimed to compare the prevalence of HSV-2 infection as the main cause of genital Herpes among two groups of women with high risk behaviors and women from normal population attending obstetrics and gynecology clinics of Tehran, Capital of Iran.

## Materials and Methods

Three hundreds and sixty two women aged 15-49 years (reproductive age) attending obstetrics and gynecology clinics of social security insurance in different districts of Tehran as low risk group and 156 prisoner women and women attending drop in center (DIC) in Tehran as high risk group, were enrolled in this cross sectional study after signing a written informed consent, which was approved by Avicenna Medical Ethics Research Committee.

Samples were collected using consecutive method in a laboratory by a person who was trained on interviewing techniques and sample collection. About 3-5 ml blood sample was taken from each participant and with completed questionnaires and informed consents transferred to Avicenna Research Institute daily.

Blood samples were centrifuged at 3000 RPM for 5-10 min to separate the blood cell mass from the serum. The sera were aliquoted into 0.5 ml and 1.5 ml vials and frozen at -20° C until use. HSV-2 antibodies were tested with Enzyme-Linked Immunosorbent Assay (ELISA) using Anti HSV-2 (IgG/IgM) ELISA Kit (Euro immune Deutschland, Germany). The serum samples were diluted 1:101 with sample buffer and the ELISA procedure was performed according to manufacturer's instruction.

According to the kit manual, the values less than 0.8 were considered as negative while equal and more than 1.1 were positive, the borderline ranged between 0.8 to 1.1 and the cutoff point was determined as 1. The kit could detect at least 0.05 anti-HSV-2 with 100% specificity.

Test results and questionnaire data were analyzed using SPSS package (hicago, IL, USA). Independent samples T-test evaluated the association between continuous and dichotomous variables; Chi Square, the association between dichotomous variables; Pearson correlation coefficient, the correlation between continuous variables and univariate logistic regression evaluated the marital status and educational level between high risk and low risk groups and being married and educational levels were considered as the reference group. Multiple logistic regressions evaluated the effect of variables on the dependent variable. The dichotomous variable of high and low risk behaviors entered the model as a dependent variable. Pvalues <0.05 were considered as significant.

## Results

The prevalence of IgG antibody in women with high risk behaviors was significantly more than other women (26.3% versus 2.5%; p<0.001). The prevalence of IgM antibody in high risk group was less than others (3.8% versus 7.1%) but the difference was not statistically significant. Also, no significant difference was found in borderline cases between two groups. The data about two groups were shown in Table 1 and Table 2. The mean age of participants in low risk group was 32.33±8.41 years and in high risk group was 35.19±11.53 (p<0.001) and the mean age at first sexual contact in low-risk and high-risk groups were 19.83±4.23 and 15.81±4.02 years (p<0.001), respectively.

The number of widowed and divorced women in high risk group was significantly more than low risk group and in contrast, the number of single and married women in low risk group was more than high risk group (p<0.001). The number of women with primary, intermediate and advanced education in low risk group was more than high risk group while illiterate women in high risk group were more than the other group (p<0.001). There was no significant difference in occupational activities between two groups.

Among sexual active women with low risk behaviors, just one participant expressed that she had anal or oral sex, while 31.8% of sexual active women in high risk group experienced anal/oral sex (p<0.001). 2.2% of low risk women vs. 20.9% of women in high risk group had more than one partner during their life (p<0.001) and 28.7% of partners in high risk women vs 5.0% of low risk women had polygamous sexual history (p<0.001). 13.4% of married women in low risk group had used condom as contraceptive method during sexual contacts and the others used no or other contraceptive methods except than condom, on the contrary, 22.5% of sexual active women in high risk group had used condom (p<0.001). 76.9% of high risk group vs. 0.3% of low risk group were cigarette smokers and 55.1% of high risk group vs. 0.3% of low risk group were drug abuser (p<0.001). The multiple logistic regressions showed that being widow or divorced (p<0.001), lower mean age in first sex (p=0.003), having anal/oral sex (p=0.035), being married more than once by woman (p=0.004) and her partner (p<0.001), smoking (p<0.001) and drug abuse (p<0.001) in high risk group were significantly more than low risk group.

The symptoms of genital infection including genital pain, burning, itching, genital skin rashes, ulcer and dysuria in high risk group were significantly more than low risk group (p=0.002), however, no significant difference was observed in abnormal vaginal discharge and history of genital infection between two groups.

In order to find the association between high risk behaviors and infection, we analyzed each study group separately. In high risk group, statistical analysis showed that there was significant association between positive IgG test results and anal/oral sex (p<0.001), condom use (p<0.001), smoking (p=0.025), addiction (p<0.001) as well as genital pain (p=0.005), burning (p=0.026), itching (p=0.033), ulcer (p=0.033), dysuria (p=0.003), and history of genital infection (p=0.018). Association between IgM and variables were not significant.

Furthermore, in low risk group, association between positive IgM and IgG test results and risky behaviors were not significant. There was significant association between IgM and genital itching (p=0.044), rash (p=0.030), and ulcer (p=0.018).

**Table 1:** Demographic data of women with high-risk and low-risk behaviors in Tehran, 2008

**Demographic data**	**Women with high-risk behaviors**	**Women with low-risk behaviors**	***P* value**
Age	35.19±11.53	32.33±8.41	P<0.001^a^
Age at first sexual contact	15.81±4.02	19.83±4.23	P<0.001 a
Marital status			
Married	63 (41.2%)	317 (87.8%)	reference^b^
Single	24 (15.7%)	40 (11.1%)	P<0.001
Widow	44 (28.8%)	3 (0.8%)	P<0.001
Divorced	22 (14.4%)	1 (0.3%)	P<0.001
Educational status			
Illiterate	44 (28.2%)	12 (3.3%)	P<0.001^b^
Elementary	79 (50.6%)	131 (36.2%)	P<0.001
Intermediate	31 (19.9%)	165 (45.6%)	P=0.033
Advance	2 (1.3%)	54 (14.9%)	reference
Occupational status			
Employed	18 (12.2%)	37 (11.2%)	P=0.113 c
Unemployed	130 (78.8%)	292 (88.8%)	
Anal/oral sex	45 (31.8%)	1 (0.3%)	P<0.001 c
Being married more than once	31 (20.9%)	7 (2.2%)	P<0.001 c
history of polygamous in partner	44 (28.7%)	18 (5.0%)	P<0.001 c
Condom use	29 (22.5%)	43 (13.4%)	P<0.001 c
Smoking	120 (76.9%)	1 (0.3%)	P<0.001 c
Drug abuse	86 (55.1%)	1 (0.3%)	P<0.001 c

^a^ Based on independent T test

^b^ Based on univariate logistic regression

^c^ Based on Chi square

**Table 2:** Infectious data of women with high-risk and low-risk behaviors in Tehran, 2008

**Infectious data**	**Women with high-risk behaviors No. (%)**	**Women with low-risk behaviorsNo. (%)**	***P* value**
Genital pain	47 (30.1)	42 (11.6)	P<0.001 a
Genital burning	51 (32.7)	68 (18.8)	P<0.001 a
Genital itching	49 (31.4)	59 (16.3)	P<0.001 a
Genital skin rashes	18 (11.5)	10 (2.8)	P<0.001 a
Genital ulcer	27 (17.3)	8 (2.2)	P<0.001 a
Dysuria	45 (28.8)	62 (17.1)	P<0.001 a
Genital discharge	60 (38.5)	135 (37.3)	P=0.843 a
History of genital infection	73 (46.8)	197 (54.7)	P=0.104 a
IgG			
Negative	112 (71.8)	344 (97.2)	reference b
Positive	41 (26.3)	9 (2.5)	P<0.001
Borderline	3 (1.9)	1 (0.3)	P=0.056
IgM			
Negative	143 (91.7)	320 (90.4)	reference b
Positive	6 (3.8)	25 (7.1)	P=0.341
Borderline	7 (4.5)	9 (2.5)	P=0.792

^a^ Based on Chi square,

^b^ Based on univariate logistic regression

## Discussion

In this study, the prevalence of HSV-2 IgG in women with high risk and low risk behaviors was 26.3% and 2.5%, respectively. The prevalence of infection among general population in developing Asian countries has been reported 10-30%.[20] A review article has reported that the seroepidemiology of infection in Asian countries among non-high risk adult ranged between 7.9- 29% and among high risk women varied between 63-80%. But these data are related to commercial sex workers.[16] The prevalence of infection in India and Thailand among patients attending to STI clinics and female sex workers has been reported 26% and 82%, respectively[21]. It seems that the prevalence of infection in high risk group in current study is lower than other Asian studies which can be caused by cultural differences or different definition of high risk group in these studies. Also, the seroepidemiology of infection in developed countries has been investigated. A study carried out in the UK has reported that the prevalence of anti-HSV-2 antibodies in women attending STI clinics was 21%[22]. Considering to the type of patients' admission to European clinics, approximate similarity in HSV-2 infection rate between our high risk population and British study is justified.

In a Switzerland study, the incidence of HSV-2 IgG in adults aged 35-46 years was 19.3%.[23] The studied population was general population who revealed much more prevalence than the current study. This disagreement may be caused by moral and religious commitment, different sample size, distinct and accurate diagnostic methods.

The rate of anti-Herpes simplex virus (HSV) antibodies has been evaluated in 132 Australian prisoner women and 58% of them showed positive results[24] which was much more than our findings and a significant association was reported between HSV-2 infection and multi-partner sex. High risk samples in our study were taken from women in DIC and prison. A group of Persian prisoner women were imprisoned because of financial sanctions and most of them were married and monogamous.

The high prevalence of IgG and low prevalence rate of IgM in high risk women may possibly be caused by sex at young ages (9 years). The mean age of women when entering the study was 35.19 years. According to the seroconversion time of infection, the discrepancy between the prevalence of IgG and IgM antibodies was justified.

Such as others in our study, test results were correlated significantly to smoking, and drug abuse.[25] In high risk group that the frequency of positive IgG test was higher than low risk group, also the prevalence of condom usage was more. Since serologic tests were positive in all the ways of transmissions, it shows that genital Herpes can be transmitted through the orolabial.[1] Therefore, there was a statistically significant relationship between the result of this test and the transmission way of oral/anal. On the other hand, the high risk group was mostly multi-partner and their IgG positive test which is a sign of infection in the past showed a significant relationship with condom use, it is likely that infection occurred earlier in unprotected sex.

On account of confidence in partner, only 22.5% of high risk group used condom as a contraceptive in their sexual intercourse but condom has been used in low risk group less than prisoners and DIC women. Perhaps women with low risk behaviors felt safe in sexual contact with their partner. Moreover, no significant correlation was identified between condom use and HSV-2 infection in low risk group. A study performed in Gambia showed that 36% of married women (at least once) were infected with genital Herpes and due to lack of protection in sexual activity; disease transmission has mostly occurred after marriage.[26]

The only executive problem during the present study was the strict coordination with prisons which reduced the sample size in high risk group. Regarding the method of sample collection, the possibility of generalizing these results about public is limited; though considering the dispersion and wide coverage of social security insurance, on the other side, highrisk samples obtained from prison and one of the DICs in Tehran, the results of this study might be applied in health plans.

In conclusion, HSV-2 infection was associated with high risk behaviors such as anal and oral sex, condom use during sexual intercourses, smoking and drug abuse. Therefore, relatively high prevalence of genital Herpes among high risk women necessitates regular screening programs. Implementation of genital Herpes screening has been recommended to begin in high-risk groups with inexpensive serological tests, especially that the majority of them were in contact with various people and the disease could be easily spread in the community. Moreover, regarding the prevalence rate of 7.1% for IgM antibody in low risk women, risk of acute infection in this group should not be ignored. Therefore, safe sex education programs and diagnosis of HSV-2 symptoms should be considered in both groups to prevent the disease transmission to others in the community. Teaching individuals for protective strategies such as condom use and avoiding multi-partner sex should be started at young ages because age at first sexual contact was 9 years old in high risk group. In addition, administration of education programs in just married couples based on the assumption that in Iranian population it is not usual to have sexual contact before marriage may have an effective role in control and prevention of HSV-2 infection.
